# Impact of mHealth on Postoperative Quality of Life, Self-Management, and Dysfunction in Patients With Oral and Maxillofacial Tumors: Nonrandomized Controlled Trial

**DOI:** 10.2196/59926

**Published:** 2025-06-25

**Authors:** Yufei Li, Yueping Wang, Yifan Wu, Hong Yu, Hua Yao, Yuqun Wang, Yulan Yin, Lan Wang, Lili Hou

**Affiliations:** 1 School of Nursing, Shanghai JiaoTong University, Shanghai, China; 2 Department of Nursing, Shanghai Ninth People's Hospital, Shanghai Jiaotong University School of Medicine, Bldg 8, 2nd Fl, 639 Manufacturing Bureau Road, Huangpu district, Shanghai, China, 86 13816033620; 3 Department of Nursing, Anhui Stomatological Hospital, Anhui Medical University, Hefei, China; 4 Department of Oral & Maxillofacial Head & Neck Oncology, Shanghai Ninth People’s Hospital, Huangpu Branch, Shanghai Ninth People’s Hospital, Shanghai, China; 5 Department of Nursing, Nanjing Stomatological Hospital, Nanjing Medical University, Nanjing, China; 6 Department of Oral & Maxillofacial Head & Neck Oncology, Shanghai Ninth People’s Hospital, Fengcheng Branch, Shanghai Ninth People’s Hospital, Shanghai, China; 7 Department of Head and Neck Oncology, Zhejiang Cancer Hospital, Hangzhou, China

**Keywords:** head and neck cancer, quality of life, self-management efficiency, mHealth, functional rehabilitation, maxillofacial tumor, postoperative dysfunction, artifical intelligence

## Abstract

**Background:**

With a focus on postoperative dysfunctions that may occur after maxillofacial tumor resection and the difficulties faced during home rehabilitation, we developed a mobile health app based on nurse-patient cooperation. The app extends rehabilitation care from hospital to home with the help of artificial intelligence, Internet of Things, and other technologies, thus helping patients to better carry out their home functional rehabilitation and meet their health needs.

**Objective:**

The primary objective of this quasi-experimental study is to evaluate the impact of the Intelligent Home Rehabilitation Care Platform on the quality of life, self-management, and functional impairment in patients with oral and maxillofacial head and neck tumors. We aim to determine whether the intervention through this platform can lead to significant improvements in these areas compared with traditional postdischarge care methods.

**Methods:**

In this study, patients with oral and maxillofacial head and neck tumors who had undergone surgery were recruited from allied hospitals in the Yangtze River Delta region, divided into an experimental group (n=138) and a control group (n=123), and received either the Intelligent Home Rehabilitation Care Platform intervention or the conventional health care and guidance, respectively. The intervention lasted 3 months, and the patients’ quality of life, self-management efficacy, and improvement in dysfunction were assessed at 1 week (baseline), 1 month (T1), and 3 months (T2) postoperatively. SPSS software (IBM Corp) was used to perform the chi-square test, rank sum test, *t* test, repeated-measures ANOVA, and generalized estimation equation for data analysis.

**Results:**

We analyzed the effects of the quality of life and self-management using the generalized estimating equation method. The generalized estimating equation results showed that after adjusting for age, sex, pathological histology, cancer stage, and primary site, the intervention group had a significantly higher improvement in quality of life than the control group at the T2 (regression coefficient, β=−68.020, 95 % CI −116.639 to −19.412; *P*=.006) stage. The degree of improvement in self-management efficacy was significantly higher in the T1 (regression coefficient, β =−7.030, 95 % CI −9.540 to −4.520; *P*<.001) and T2 (regression coefficient, β =−13.245, 95 % CI −16.923 to −9.566; *P*<.001) stages than in the control group. The results of repeated-measures ANOVA and rank sum test showed that the experimental group showed improvements in shoulder function, dysphagia, and trismus after mHealth intervention; however, the differences were not significant.

**Conclusions:**

The Intelligent Home Rehabilitation Care Platform interventions can effectively enhance patients’ self-management efficacy, improving quality of life and facilitate recovery from dysfunctions. Therefore, mHealth may be used in oncology care to provide smarter home self-care for patients.

## Introduction

### Background

Oral and maxillofacial head and neck malignant tumors are highly fatal and among the top 10 malignant tumors worldwide, accounting for approximately 7% of systemic malignant tumors, with approximately 300,000 new cases and 128,000 related deaths yearly, of which oral cancer is the most common cause [1,2]. The 2024 American Cancer Society’s Cancer Statistics report revealed that the incidence of oral and pharyngeal cancers has increased by an average of 1% per year, and the mortality rate from human papillomavirus–related oral cancers has shown an increase of 2% per year [3].

The treatment and care of oral and maxillofacial head and neck tumors is a comprehensive process that includes several aspects, such as surgical treatment, radiotherapy, chemotherapy, and supportive care; surgical treatment is currently the most dominant and effective method of treating oral and maxillofacial head and neck tumors [4]. The surgical approach and surgical area involved in oral and maxillofacial head and neck tumor treatment are more complex and extensive, and surgical resection inevitably damages parts of the normal tissues. In addition to the specificity of the disease, patients often experience various complications and physiological dysfunctions in the postoperative period, such as swallowing difficulties, shoulder dysfunction, and trismus, which greatly affect survival quality [5].

Therefore, it is particularly important to provide comprehensive management to patients as it can improve their functional disorders and quality of life. During hospitalization, a professional nursing team provides patients with full care and early guidance on functional rehabilitation exercises. However, functional rehabilitation is a long-term process, and studies have shown that it usually takes 3‐6 months to recover from the symptoms and dysfunctions that occur in patients with head and neck cancer postoperatively. Therefore, patients need to continue home-based self-exercise to promote functional rehabilitation after discharge from the hospital [6]. However, some studies have shown that patients are deprived of systematic care management services after being discharged due to time and space requirements of traditional follow-ups. Notably, most patients are not well equipped to continue self-management and functional rehabilitation training in the home environment because of their lack of medical background and knowledge and poor knowledge of predischarge health education, resulting in a prolonged recovery period, poor rehabilitation effect, and high readmission rate, greatly affecting postoperative survival quality [7-9].

With the recent development and advancement of mHealth, mHealth software has been increasingly applied in the self-management and functional rehabilitation of patients with cancer and chronic disease [10]. Mobile health care supports medical and public health practices using mobile devices, such as mobile phones, personal digital assistants, patient-monitoring devices, and other wireless devices [11]. Mobile health care establishes a more convenient, comprehensive, and detailed service and communication bridge between doctors, nursing staff, and patients in hospitals, communities, and at home, which further promotes and develops the continuity of care services and, to some extent, facilitates patient self-monitoring, management, and treatment adherence; however, it is difficult to provide objective measurements based only on the software level. The popular application and technological development of the Internet of Things (IoT) also provide a boost for more convenient, accurate, and objective remote collection of medical data [12]. IoT literally means interconnected network of physical objects or “Things” integrated to exchange data between devices and systems using the web. In this regard, the Internet of Medical Things, on the other hand, is a specific app of IoT in the medical field, where remote patient monitoring, screening, and treatment are realized through wearables, sensors, and other devices telemedicine, which has been successfully adopted by caregivers or health care providers and patients [13].

### The Study

Patients with oral and maxillofacial tumors need self-management and functional rehabilitation at home after operation [5], but at present, there is insufficient coordination between China Medical Union and community rehabilitation centers, which leads to the lack of professional guidance at home [14]. Most of the existing mobile medical apps support only one-way information transmission and mainly rely on patients to fill in data subjectively, which makes it difficult to guarantee the accuracy of data and seriously affects the user experience and compliance of patients [15]. Therefore, this study developed an intelligent home rehabilitation care platform integrating software and IoT equipment, aiming at expanding the scope of nursing, providing convenience for nurse-patient communication through monitoring and recording medical data, and helping patients recover after operation. The platform adopts the professional nursing process of “Assessment-Planning-Implementation-Evaluation” to help patients recover their functions and improve their quality of life. To the best of our knowledge, there is no similar comprehensive rehabilitation care platform for postoperative rehabilitation of patients with oral and maxillofacial tumors. The goal of this study is to guide and supervise patients’ rehabilitation through mobile medical technology, enhance their self-management ability, and reduce the incidence of family adverse events.

## Methods

### Study Design and Sample

This is a quasi-experimental study of a multicenter collaboration. Four hospitals conducted the data collection. The ethical approval process and trial registration were completed before the first patient was formally enrolled in the study. The inclusion criteria were age ≥18 years; basic cognitive ability; history of oral and maxillofacial head and neck tumors (tumors originating from the oral cavity, oropharynx, hypopharynx, larynx, and other regions of the head and neck) treated with surgery only; tumor, node, and metastasis clinical stage I-III; absence of distant metastases; and at least one of the following postoperative dysfunction complications or other special care needs: dysphagia, trismus, malnutrition, shoulder dysfunction, or having been discharged with a tracheostomy tube after tracheotomy. The exclusion criteria included the presence of psychiatric disorders or cognitive dysfunction, comorbid serious cardiovascular and cerebrovascular underlying diseases (eg, cardiac infarction, cerebral infarction, and coronary artery disease), pregnancy, and other cancers or severe comorbidities.

### Features of the Intelligent Home Rehabilitation Platform

The Intelligent Home Rehabilitation Care Platform developed in this study comprised 2 parts: an Android-based mobile app and Bluetooth-connected wireless IoT devices. The software allowed login access through 2 separate portals. The web-based port was available for health care professionals to log in. After logging in, health care professionals could execute modules such as patient screening and inclusion, symptom assessment, plan development, scale management, communication channels, and exception handling on the platform to develop a personalized home rehabilitation plan for patients. Each patient was given a free-to-use smart tablet before discharge (to be returned at the end of the intervention period) and logged in to the app software port on the tablet through a personal account added by the system administrator. The digital platform was intended for use with study participants only, as a tool for data collection and interaction with study participants, and did not involve any form of participant monitoring beyond the parameters of the study. After logging in, patients could access medical and health care knowledge, view and execute plans, and contact health care professionals. The platform had a suite of outreach devices, including a wireless oximeter with Bluetooth connectivity and a food scale, which can automatically collect and upload the patient’s oxygen saturation metrics and daily nutritional intake.

The app software part of the platform contained 6 modules: rehabilitation calendar, training video upload, message reminder, communication channel, health promotion media library, and follow-up scale filling, which could provide 5 major home rehabilitation nursing guidelines and training: mouth opening, swallowing, shoulder exercise, nutritional intake, and tracheotomy. According to different home rehabilitation nursing needs, we developed, designed, and filmed the corresponding professional training guidance videos, which patients could follow for home rehabilitation exercises and upload according to their personalized plan schedule created by health care professionals through the platform and configured IoT devices. We also built a media database of health knowledge containing 20 live actions, animated home rehabilitation, and nursing videos for patients to watch. In addition, the platform had a communication channel through which patients could directly establish text or voice contact with health care personnel in case of emergency. Furthermore, the developed Intelligent Home Rehabilitation Care Platform was an integrated platform that could be jointly used by the Yangtze River Delta Consortium units. An additional figure file showed screenshots of the different ports on the platform (Multimedia Appendix 1).

### Development, Training, and Implementation of the Intervention

The study consisted of a total of 43 multidisciplinary experts and clinical caregivers from 4 hospitals, including clinical surgeons, nutritional managers, rehabilitation therapists, nurses, and so forth. All mHealth-based interventions were constructed based on evidence-based results from the project team’s previous work [16]. For example, the diet and nutrition program included recipes for total nutrient solution nasal feeding, nutritional recipes for patients on a normal diet, timing of implementation of nutrition, nutritional pathways, and nutritional energy and types; swallowing included dimensions such as oral muscle training methods and airway protection maneuvers; and the rehabilitative care program for shoulder exercise included dimensions such as passive exercise, active exercise, frequency of exercise, and duration of exercise.

Before the platform was officially used, several rounds of centralized training and guidance on its use were provided to the project implementation members. Each patient will complete a functional assessment and quality-of-life and self-efficacy survey on a one-to-one basis prior to discharge. The control and experimental groups received routine health education, home rehabilitation nursing guidance, and rehabilitation plan suggestions before discharge, whereas the experimental group further received 30 minutes of additional guidance via the mHealth platform. The extra services included helping participants and their caregivers recognize and use the Intelligent Home Rehabilitation Care Platform and its outreach smart devices and explaining the interventions that need to be completed when using the platform at home: the implementation of an intervention plan individually tailored to them.

The intervention period was for 3 months. At each follow-up node, when the patient’s dysfunctions had returned to a normal level, the rehabilitation program was terminated, but the other platform functions continued to be used until the end of the 3-month period.

In this paper, we focus on the impact of mHealth on postoperative quality of life, self-management efficacy, and functional improvement of shoulder function, dysphagia, and trigeminal neuralgia in patients with oral and maxillofacial tumors. The analysis of nutritional improvement and tracheotomy care and the effects and economic benefits of the Intelligent Home Rehabilitation Care Platform will be elaborated in detail in a subsequent paper.

### Data Collection

We conducted clinical data collection in 12 oral and maxillofacial head and neck tumor wards in 4 regional units of the Yangtze River Delta Consortium. To minimize the interference of confounding factors in the design of nonrandomized controlled study, and considering the resource allocation and total number of patients in different wards in multicenter, wards were naturally allocated. Different wards are not allocated according to disease risk level or tumor-site preference. To be considered for the symptom management program and rehabilitation strategies we offered, patients were required to have 1 or more of the following postoperative complications at the sample inclusion stage: malnutrition, dysphagia, shoulder dysfunction, trismus, or having been discharged with a tube after tracheotomy. For patients with multiple symptoms, we established a corresponding intervention plan for each symptom and included them in different symptomatic subgroups that were analyzed at the same time.

Before discharge, each patient was individually instructed by the researcher on disease-related health knowledge, postdischarge functional rehabilitation exercise methods and points, airway home care, and follow-up review reminders. In this study, the data collected from patients at 1 week postoperatively (T0) were used as baseline data, and electronic questionnaires were used to investigate the outcome of the intervention in the 2 patient groups at the first (T1) and third (T2) postoperative months.

### Questionnaires

The questionnaire used in this study was a general information questionnaire that included questions about patients’ demographic characteristics (age, sex, marital status, and education level), clinical characteristics (cancer stage, primary site of cancer, and days of hospitalization), and the University of Washington Quality of Life Scale (UW-QOL; version 4), which is a patient-specific quality-of-life questionnaire for head and neck cancers [17]. The UW-QOL consists of 15 items with 12 disease-specific questions on pain, appearance, activity, recreation, swallowing, chewing, speech, shoulder, taste, saliva, mood, and anxiety, and 3 composite questions. The scale has been translated into several versions, and the Chinese version also has good reliability, with a Cronbach coefficient of 0.90 for the UW-QOL. Strategies Used by People to Promote Health (SUPPH) was used to assess patients’ self-management efficacy [18]. The Chinese version of the SUPPH scale consists of 28 items in the areas of self-determination, stress relief, and positive attitude. The SUPPH evaluation index is calculated as (the actual score on the scale/the total score on the scale) × 100%, with ≥80%, 61%‐79%, and ≤60% categorized as high, medium, and low levels, respectively. The Cronbach coefficient of the SUPPH (Chinese version) is 0.97. All patients were assessed using the UW-QOL and SUPPH scales, which was the primary outcome indicator in this study.

For symptom assessment, symptomatic patient groups were individually assessed using targeted scales. The Constant-Murley Shoulder Score was used to evaluate shoulder dysfunction, the Kubota Water Swallowing Test was used to evaluate dysphagia, and the Subjective Objective Management and Analytic criteria were used to evaluate trismus.

### Simple Size and Power

With no previous experiments from which to obtain expected significant differences or sample SDs, we conducted a small pilot trial to calculate the sample size before the start of the formal study, which collected preintervention-postintervention changes in quality-of-life and self-management efficacy scores using the UW-QOL and the SUPPH. We calculated the pooled sample SD using the pooled results, and the sample size was calculated using G*Power (version 3.1; Heinrich-Heine-Universität Düsseldorf), with test levels chosen as α=.01 and β=.1. The calculations showed that at least 104 patients should be collected in each of the experimental and control groups. Thus, at an assumed attrition rate of 20%, the final formal experimental sample sizes of the experimental group and the control group should each recruit at least 130 patients.

### Statistical Analysis

Continuous variables with normally distributed data are expressed as mean (SD), nonnormal data are expressed as median with quartiles (median, P25-P75), and categorical variables are expressed as n (%). Differences in categorical variables were tested by chi-square test, rank sum test, or Fisher exact test, and differences between groups for continuous variables at each time point were tested by independent *t* test. Differential changes in primary outcomes (quality of life and self-management) at T1 and T2 compared with T0 between the 2 groups were assessed using generalized estimating equations, and repeated-measures ANOVA was used to assess the improvement in functioning (shoulder functioning) between the 2 groups at T1 and T2. Generalized estimating equation analyses were used because the method takes into account internal correlations between repeated measures. Adjusted generalized estimating equation analyses were performed using covariates, including age, sex, pathological histology, cancer stage, and primary site. All statistical assessments were double-tested, and statistical significance was set at *P*<.05. Statistical analyses were performed using IBM SPSS (version 25.0; IBM Corp).

### Ethical Considerations

This study was approved by the Institute in the Hospital Ethics Committee (SH9H-2023-T157-1) and abided by the principles of Declaration of Helsinki. All patients who participated in the study participated voluntarily and obtained the informed consent of each patient in writing. Patients have the right to withdraw from the study at any time. Due to the principle of confidentiality, each participant’s information will be anonymized and given a code during the data analysis stage. Individuals cannot be identified in any results presentation. Additionally, no compensation was provided to study participants.

## Results

### Analytical Sample

**Figure 1.**
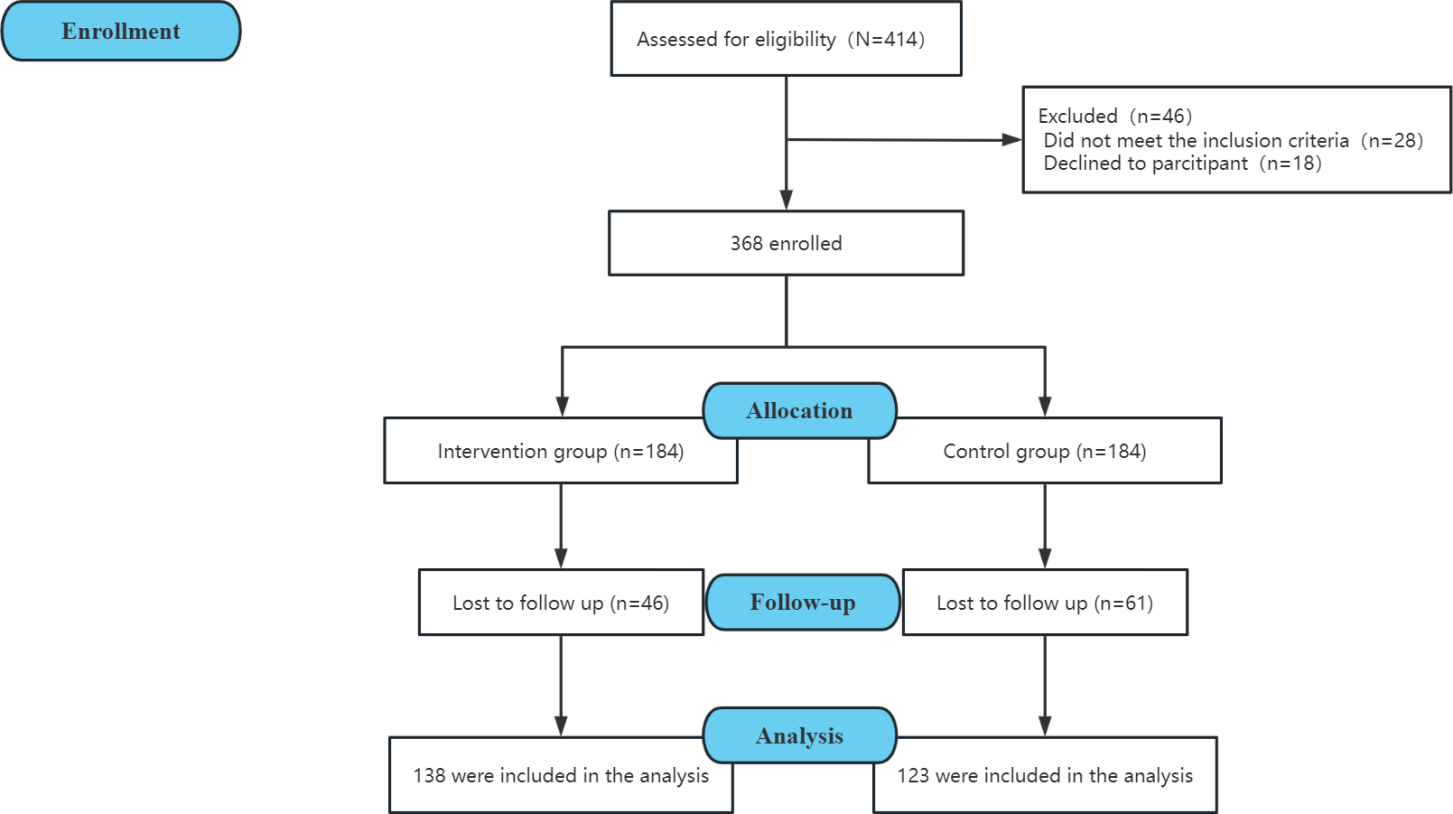
Flowchart of included participants.

A total of 368 participants (184 in the experimental group and 184 in the control group) were enrolled in this study. After a 3-month intervention period with 2 follow-up measurement time points, 25% (46/184) of patients in the experimental group and 33% (61/184) of patients in the control group were lost to visit or dislodging. The final sample included 138 and 123 patients in the experimental and control groups, respectively (Figure 1).

### Participants’ Background Characteristics

A total of 261 patients with oral and maxillofacial head and neck tumors who had undergone surgery were recruited in this study, including 162 men and 99 women, with a mean age of 54.84 (SD 14.45) years. The marital status of most patients was married (234/261, 89.7%), and the more predominant education level was middle school (82/261, 31.4%). Furthermore, most patients had stage II tumors (109/261, 41.8%), the predominant primary site of the tumor was the tongue (116/261, 44.4%), and the median length of hospitalization was 18 days (Table 1).

**Table 1.** Participants’ baseline demographics and clinical characteristics (N=261).

Variable	Total (N=261）	Experimental group (n=138）	Control group (n=123）	*P* value
Demographics				
Sex, n (%)				<.01^a^
Male	162 (62.1)	97 (70.3)	65 (52.8)	
Female	99 (37.9)	41 (29.7)	58 (47.2)	
Age (years), mean (SD)	54.84 (14.45)	55.34 (13.17)	54.28 (15.80)	.56
Marital status, n (%)				.09
Married	234 (89.7)	122 (88.4)	112 (91.1)	
Unmarried	12 (4.6)	4 (2.9)	8 (6.5)	
Other (widowed/divorced）	15 (5.7)	12 (8.7)	3 (2.4)	
Education, n (%)				.14
Below primary school	60 (23.0)	28 (20.3)	32 (26.0)	
Middle school	82 (31.4)	52 (37.7)	30 (24.4)	
High school	57 (21.8)	28 (20.3)	29 (23.6)	
Undergraduate and above	62 (23.8)	30 (21.7)	32 (26.0)	
Career status, n (%)				.58
In-service	113 (43.3)	56 (40.6)	57 (46.3)	
Separated/retired	128 (49.0)	70 (50.7)	58 (47.2)	
Unemployed	20 (7.7)	12 (8.7)	8 (6.5)	
Smoking status, n (%)				.36
Smoking	39 (14.9)	22 (15.9)	17 (13.8)	
Quit smoking	77 (29.5)	45 (32.6)	32 (26.0)	
Never smoking	145 (55.6)	71 (51.4)	74 (60.2)	
Alcohol status, n (%)				.08
Regular alcohol	27 (10.3)	15 (10.9)	12 (9.8)	
Quit alcohol	89 (34.1)	55 (39.9)	34 (27.6)	
Never had alcohol	145 (55.6)	68 (49.3)	77 (47.1)	
Clinical characteristic				
Pathological histology, n (%)				.63
Squamous cell carcinomas	209 (80.1)	112 (81.2)	97 (78.9)	
Adenoid cystic carcinoma	23 (8.8)	13 (9.4)	10 (8.1)	
Others	29 (11.1)	13 (9.4)	16 (13.0)	
Cancer stage, n (%)				.38
I	83 (31.8)	40 (29.0)	43 (35.0)	
II	109 (41.8)	63 (45.7)	46 (37.4)	
III	69 (26.4)	35 (25.4)	34 (27.6)	
Primary site, n (%)				.09
Tongue	116 (44.4)	66 (47.8)	50 (40.7)	
Buccal side	49 (18.8)	24 (17.4)	25 (20.3)	
Hard palate	16 (6.1)	7 (5.1)	9 (7.3)	
Gums	24 (9.2)	13 (9.4)	11 (8.9)	
Parotid and submaxillary glands	10 (3.8)	1 (0.7)	9 (7.3)	
Throat	17 (6.5)	12 (8.7)	5 (4.1)	
Lip	2 (0.8)	0 (0)	2 (1.6)	
Mouth floor	3 (1.2)	2 (1.5)	1 (0.9)	
Jaws	24 (9.2)	13 (9.4)	11 (8.9)	
Length of hospitalization (days), median (IQR)	18 (15-21)	19 (16-22)	18 (14-21)	.08

a *P*<.05.

### mHealth Intervention Improved Quality of Life

The UW-QOL scores of the patients are shown in Table 2; higher scores are associated with better quality of life. At baseline, the total quality-of-life scores of the experimental and control groups were 625.38 and 647.11. There were no statistical intergroup differences at baseline (mean difference −21.73, SD 21.90; 95 % CI −64.86 to 21.4; *P*=.322). However, after 1 month of intervention (T1), the total quality-of-life scores of the experimental and control groups increased to 826.38 and 875.04, respectively, and there was a significant difference in the quality of life between the 2 groups at this stage (*P*<.01). After 3 months of intervention (T2), the total quality-of-life scores of the experimental group and the control group improved to 957.88 and 911.59, respectively, and there was a significant difference between the 2 groups in terms of improvement in quality of life (*P*<.01), and the overall improvement in quality of life of the experimental group was higher than that of the control group (mean difference 46.29, SD 16.83; 95 % CI 13.14-79.44; *P*=.006).

**Table 2.** Follow-up of primary outcomes among 2 groups based on University of Washington Quality of Life Scale.

Outcome by group	T0 (baseline)			T1 (1 month)			T2 (3 months)		
	Mean (SD)	Difference between groups (MD,95% CI)	*P* value	Mean (SD)	Difference between groups (MD,95% CI)	*P* value	Mean (SD)	Difference between groups (MD,95% CI)	*P* value
Experimental group	625.38 (170.74)	−21.73, −64.86 to 21.40	.322	826.38 (129.33)	−48.66, −84.10 to −13.23	.007^a^	957.88 (911.59)	46.29, 13.14 to 79.44	.006^a^
Control group	647.11 (183.00)			875.04 (161.02)			911.59 (141.07)		

a *P*<.05.

Furthermore, the generalized estimating equation models adjusted for age, sex, pathological histology, cancer stage, and primary site showed significant interaction terms (group × time) at 3 months (β=−68.020, 95 % CI −116.639 to −19.412; *P*=.006; Table 3), which validating that Intelligent Home Rehabilitation Platform–based intervention was effective in improving patients’ quality of life.

**Table 3.** Modeling changes in the total score of University of Washington Quality of Life Scale over time by adjusting side variables using the generalized estimating equation model (N=261).

UW-QOL^a^	β	SE	95% CI		Wald χ^2^	*P* value^b^
Group	15.109	22.438	−28.869 to 59.086		0.453	.501
Time						
Baseline	Reference					
T1	201.000	14.185	173.199 to 228.801		200.795	<.001^c^
T2	332.500	15.329	302.455 to 362.545		470.474	<.001^c^
Group × time						
Group × T0	Reference					
Group × T1	26.935	23.391	−18.911 to 72.781		1.326	.250
Group × T2	−68.020	24.801	−116.639 to −19.412		7.522	.006^c^

a UW-QOL: University of Washington Quality of Life Scale.

b Adjusted with age, sex, pathological histology, cancer stage, and primary site.

c *P*<.05.

### mHealth Intervention Improved Self-Management Efficacy

Table 4 shows the statistical results of the patients’ SUPPH scores, with higher scores indicating higher self-management efficacy. At baseline, the self-management efficacy scores of the experimental and control groups were 64.51 and 65.79, respectively. There were no statistical intergroup differences at baseline (mean difference −1.274, SD 2.50; 95 % CI −6.19 to 3.64; *P*=.610). After 1 month of intervention, the self-management efficacy scores of the experimental and control groups increased to 83.54 and 77.78, respectively, and the difference between the 2 groups was significant at this stage (mean difference 5.76, SD 2.22; 95 % CI 1.38-10.13; *P*=.01). After 3 months of intervention, the self-management efficacy scores of the experimental group and the control group further improved to 101.44 and 89.47, respectively, with the experimental group’s self-management efficacy scores being significantly higher than those of the control group (mean difference 11.97, SD 2.01; 95 % CI 8.01-15.93; *P*<.001).

**Table 4.** Participants’ (N=261) Strategies Used by People to Promote Health–measured self-management efficacy scores before and after the mHealth intervention.

Outcome by group	T0 (baseline)			T1 (1 month)			T2 (3 months)		
	Mean (SD)	Difference between groups (MD,95% CI)	*P* value	Mean (SD)	Difference between groups (MD,95% CI)	*P* value	Mean (SD)	Difference between groups (MD,95% CI)	*P* value
SUPPH^a^									
Experimental group	64.51 (19.63)	−1.27, −6.19 to 3.64	.610	83.54 (18.15)	5.76,1.38 to 10.13	.01^b^	101.44 (15.61)	11.97,8.01 to 15.93	<.001^b^
Control group	65.79 (20.70)			77.78 (17.65)			89.47 (16.86)		

a SUPPH: Strategies Used by People to Promote Health.

b *P*<.05.

In addition, the interaction term (group× time) of the 1-month (β=−7.030, 95% CI −9.540 to −4.520; *P*<.001) and 3-month (β=−13.245, 95% CI −16.923 to −9.566; *P*<.001) adjusted models showed that, over time, patients who received the smart platform intervention had a self-management efficacy improved further (Table 5). These results suggest that the Intelligent Home Rehabilitation Platform is effective in improving patients’ self-management abilities during the home rehabilitation period.

**Table 5.** Modeling changes in the total score of Strategies Used by People to Promote Health over time by adjusting side variables using the generalized estimating equation model (N=261).

SUPPH^a^	β	SE	95% CI		Wald χ^2^	*P* value^b^
Group	.610	2.547	−4.381 to 5.601		0.057	.811
Time						
Baseline	Reference					
T1	19.022	0.930	17.200 to 20.844		418.706	<.001^**c**^
T2	36.928	1.376	34.231 to 39.625		720.181	<.001^c^
Group × time						
Group × T0	Reference					
Group × T1	−7.030	1.281	−9.540 to −4.520		30.132	<.001^c^
Group × T2	−13.245	1.877	−16.923 to −9.566		49.799	<.001^c^

a SUPPH: Strategies Used by People to Promote Health.

b Adjusted with age, sex, pathological histology, cancer stage, and primary site.

c *P*<.05.

### mHealth Intervention Improved Dysfunctions

To assess patients’ symptoms and develop a personalized intervention plan, we additionally assessed the patients with dysfunctions (reduced shoulder mobility, dysphagia, and trismus) in both groups at baseline and, based on the assessment results, we developed a rehabilitation program for these patients and evaluated them using the same measurement tools at 1 and 3 months postoperatively to confirm the effectiveness of the platform intervention. Tables S1-S5 in Multimedia Appendix 2 present the data on improvements in shoulder function, swallowing function, and mouth opening in both groups after the Intelligent Home Rehabilitation Care Platform intervention.

#### Shoulder Function

Among the recruited patients, 24 patients who developed shoulder dysfunction in the postoperative period were included (13 in the experimental group and 11 in the control group). Shoulder function was scored on a total point scale, with higher total scores associated with better shoulder function. At baseline, the mean shoulder function scores of the experimental and control groups were 60.77 and 55.64, respectively, with no statistical difference between the 2 groups. After 1 month of intervention, the mean shoulder function scores of the experimental and control groups increased to 76.38 and 70.09, respectively; after 3 months of intervention, the mean shoulder function scores of the experimental and control groups further increased to 84.46 and 77.45, respectively. There was no significant difference in the functional status of the shoulder joint between the 2 groups at the 2 measurement points. Repeated-measures ANOVA results showed that the 2 groups were statistically different in terms of the time effect at *P*<.001, indicating that the change in shoulder function scores over time at each time point was statistically different.Detailed results are shown in Table S1 in Multimedia Appendix 2.

#### Dysphagia

Among the recruited patients, 43 patients who developed dysphagia in the postoperative period were included (19 in the experimental group and 24 in the control group). The swallowing test was assessed on a scale of 5 grades: I-V, where the smaller the grade, the better the swallowing function. In the evaluation of outcome indicators, “Resolved” indicated that dysphasia had disappeared, and the level of the water-swallowing test was classified as grade I; “Significantly effective” indicated that dysphasia had significantly improved, and the level of the water-swallowing test was increased by 2 grades; “Improved” indicated that dysphasia had improved, and the level of the water-swallowing test was increased by 1 grade; and “Ineffective” indicated that the improvement of dysphasia was insignificant, and the level of the water-swallowing test was unchanged. These improvement grades were considered effective.

At baseline, patients in the experimental group and the control group had different degrees of dysphagia, with no significant difference between the 2 groups. After 1 month of intervention, the water-swallowing test grades of patients in the control and experimental groups improved. After 3 months of intervention, most patients were resolved, which was a significant improvement from baseline. Treatment effectiveness rates in the experimental and control groups were 94.74% and 87.50%, respectively. There was no significant difference in the effect of treatment on swallowing function between the 2 groups; however, the overall effectiveness rate in the experimental group was higher than that in the control group, and the specific data are shown in detail in Tables S2 and S3 in Multimedia Appendix 2.

#### Trismus

Among the recruited patients, 73 patients who developed postoperative trismus were included (38 in the experimental group and 35 in the control group) . The degree of trismus was evaluated according to the Subjective Objective Management and Analytic criteria: 0 indicated normal mouth opening, with an incisor distance of >3.5 cm; grade I indicated restricted mouth opening, with an incisor distance of 2.1‐3.0 cm; grade II indicated difficulty in dry food intake, with an incisor distance of 1.1‐2.0 cm; grade III indicated difficulty in soft food intake, with an incisor distance of 0.5‐1.0 cm; and grade IV indicated the need for nasal feeding, with an incisor distance of <0.5 cm. The higher the grade, the more severe the degree of trismus. Regarding efficacy evaluation, the same criteria as that used in the dysphagia component were used to evaluate efficacy.

At baseline, patients in the experimental and control groups had grades I-III trismus, with no significant difference between the 2 groups. After 1 month of intervention, both the control and experimental groups showed improvement in the degree of trismus. After 3 months of intervention,>50% of the patients were resolved, which was a significant improvement from baseline. The treatment effectiveness in the experimental and control groups was 84.21% and 91.43%, respectively. The effect of treatment on trismus in the 2 groups was not significant between the 2 groups, as shown in Tables S4 and S5 in Multimedia Appendix 2.

## Discussion

### Principal Findings

Previous research has shown that patients with oral and maxillofacial head and neck tumors experience deterioration in their health status and symptoms during the acute treatment phase within 3 months. This deterioration may be attributed to the adverse effects of treatments such as surgery and radiotherapy. However, symptoms tend to improve and gradually stabilize between 3 and 6 months after treatment [6]. A prospective study revealed that physiological status (fatigue, pain, nausea, and vomiting) and physical activity (resuming leisure activities and sustainable work) significantly worsened within the first 3 months after oral cancer surgery [19]. Postoperatively, patients experience a significant decline in social functioning, body image, and financial status. Therefore, we administered an intervention using the Intelligent Home Rehabilitation Care Platform to address these challenges within the first 3 months postoperatively, and the results indicate a significant improvement in shoulder function, dysphagia, and trismus in patients after 3 months postoperatively, with some returning to normal function. These findings were consistent with those of a previous study [6].

Reports suggest that the quality of life of survivors of head and neck cancer is generally lower during different postoperative periods, affecting their survival rate [20,21]. This may be due to disease-related and treatment-related factors, leading to physiological impairment and body image issues [22]. Symptom control and functional improvement are long-term tasks. Health care professionals typically provide health education through verbal instructions or knowledge manuals upon discharge; however, patients often have limited understanding of and compliance with these methods and lack medical and health knowledge [8,23-25]. Therefore, mobile health care provides a solution for improving patients’ access to medical knowledge and providing scientific guidance for family rehabilitation.

Previous studies explored the impact of different forms of mobile health care on improving quality of life, care needs, psychological and physiological functions, and other aspects of patients with head and neck cancer [26]. However, these mobile health care forms are primarily mobile apps, websites, or other wearable devices, and the involvement of health care professionals is limited. In contrast, the developed Intelligent Home Rehabilitation Care Platform in this study closely connects patients with health care professionals. Before discharge, health care professionals assess the patients and personalize home-based rehabilitation training plans based on each patient’s symptoms and characteristics. Patients follow these plans and receive reminders when they forget. Furthermore, patients can obtain medical and health knowledge and contact health care professionals on the platform, which, to a certain extent, also increases patients’ adherence to rehabilitation training and helps them build up their confidence and self-management ability.

This study’s results showed that using the Intelligent Home Rehabilitation Care Platform significantly improved patients’ quality of life at 3 months postoperatively compared with the baseline level; however, such difference was not observed at 1 month postoperatively, possibly due to the shorter duration of the intervention. There was a difference in the improvement of quality of life between the treatment and control groups at 3 months postoperatively, suggesting that the effect of the intervention becomes progressively more significant as the intervention time increases.

We also obtained better feedback on self-management efficacy, which was not significantly different between the 2 groups at baseline, and was evaluated based on the SUPPH scores. We observed that self-management efficacy was low in both groups at baseline, possibly because the patients had just received surgical treatment, experienced physiological discomfort, and had somatic limitations. At 1 month postoperatively, the self-management efficacy scores in both groups improved; however, the overall score was still low. At 3 months postoperatively, the self-management efficacy of both groups improved significantly, with the efficacy scores in the control group increasing to a medium level and that in the experimental group increasing to a high level, suggesting that mHealth interventions can significantly increase patients’ self-management information and efficacy, which has not been reported in previous studies.

Shoulder dysfunction is usually due to functional impairment caused by clearance of the operative area, and studies have shown that adherence to functional shoulder exercises can effectively improve postoperative shoulder dysfunction [27]. We developed a comprehensive rehabilitation program for patients with shoulder dysfunction to improve shoulder function through pendulum exercises and resistance training. In this study, there was no significant difference in the shoulder functional status between the 2 groups at the 2 measurement points. Overall, the experimental group demonstrated better improvements than the control group. Similarly, we did not observe significant differences in the results of dysphagia and trismus measurements; however, the overall efficacy rate in the experimental group was higher than that in the control group, possibly due to the small sample size of this study. In addition, the objective indicators did not demonstrate statistically significant differences compared with the subjective indicators, possibly indicating that the intervention implemented in this study was more effective at the level of subjective perception improvement compared with the objective measures.

The results of this study are important for guiding the postoperative care of patients with oral and maxillofacial tumors. The implementation of the Smart Home Rehabilitation Nursing Platform demonstrates that it has the potential to greatly enhance patient self-management and improve quality of life during the critical postoperative period. By using mHealth technology, the platform provides a new way to extend rehabilitation care from the hospital to the home environment, thereby bridging a critical gap in the continuum of care. The combination of artificial intelligence and the IoT makes remote collection of health care data easier, more accurate, and objective, thereby informing personalized care plans and facilitating timely medical interventions. In addition, the platform provides patients with accessible medical knowledge and ongoing support from health care professionals, which may increase patient adherence to rehabilitation programs and improve outcomes. Therefore, we believe that the adoption of this technology could change the traditional model of postoperative care by emphasizing the importance of patient-centered technology–assisted rehabilitation strategies in improving patient autonomy and overall well-being.

### Limitations and Recommendations

This study has some limitations. First, this study did not use a rigorous randomized controlled trial design and did not set up a blind method, which may lead to confounding factors such as placebo effect, so we need to use randomized clinical trials to further verify our results in the future. Second, in practice, patients do not present with symptoms individually but usually in clusters; however, in this study, the strategy for symptom management was to intervene separately for multiple symptoms, which may have increased the workload of health care professionals and limited the efficiency of symptom management. Third, owing to time constraints, we implemented only a 3-month follow-up intervention and did not continue the follow-up to a longer time point after the intervention period; although we achieved relatively positive results, there is a need to expand the sample size as well as validate the impact of the intervention on long-term efficacy and quality of life in the future through larger-scale replication and longer follow-up interventions. In addition, the attrition rate of both groups in this study is high. Although the potential impact has been reduced as much as possible by expanding recruitment, future research should further optimize the research process and improve the platform experience to better cope with the challenge of sample loss. Finally, this study involved multicenter data collection in the Yangtze River Delta region of China, and the results may not apply to patients with oral and maxillofacial head and neck tumors in other regions (western or northern regions).

### Conclusions

This study’s results suggest that compared with conventional follow-up measures, interventions using the Intelligent Home Rehabilitation Care Platform can effectively enhance patients’ self-management efficacy, promoting dysfunctional rehabilitation and improving postoperative survival quality.

## Supplementary material

10.2196/59926 Multimedia Appendix 1 Screenshots of the interfaces of the app.

10.2196/59926 Multimedia Appendix 2 Data on improvements in shoulder function, swallowing function, and mouth opening.

10.2196/59926 Checklist 1 CONSORT-EHEALTH checklist.

## Abbreviations

IoT: Internet of Things
SUPPH: Strategies Used by People to Promote Health
UW-QOL: University of Washington Quality of Life Scale
