# Supplementary material for: Impact of mHealth on Postoperative Quality of Life, Self-Management, and Dysfunction in Patients With Oral and Maxillofacial Tumors: Nonrandomized Controlled Trial
Source: JMIR Mhealth Uhealth. 2025 Jun 25;13:e59926. doi: 10.2196/59926 (PMC12242703; doi:10.2196/59926)

Intelligent Home Rehabilitation Care Platform

# Web Login Port (For medical Staff)

- **Login screen**


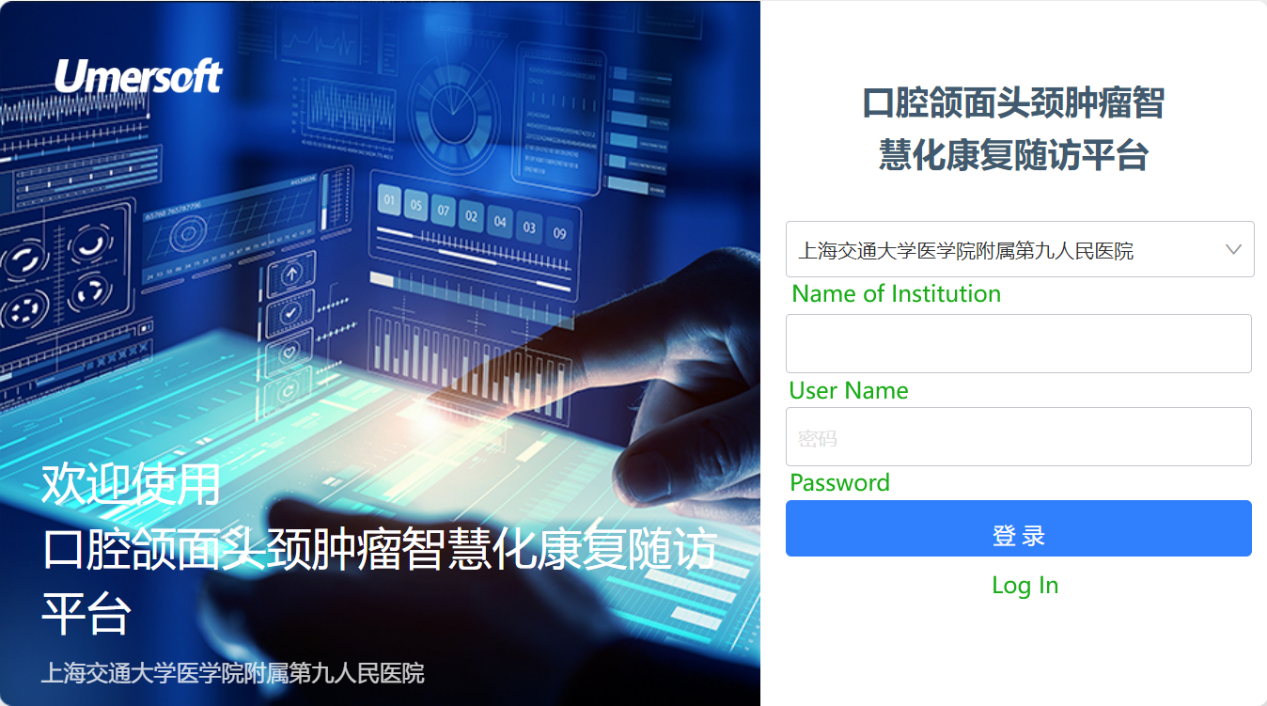


- **Main interface**


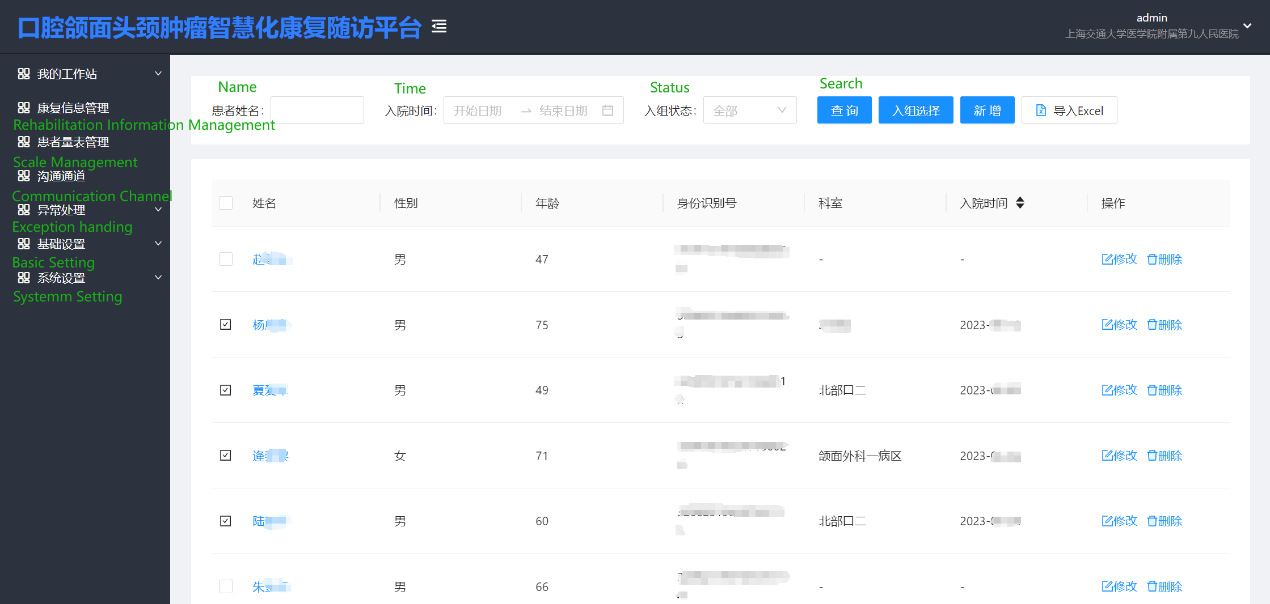


- **Assessment**

**
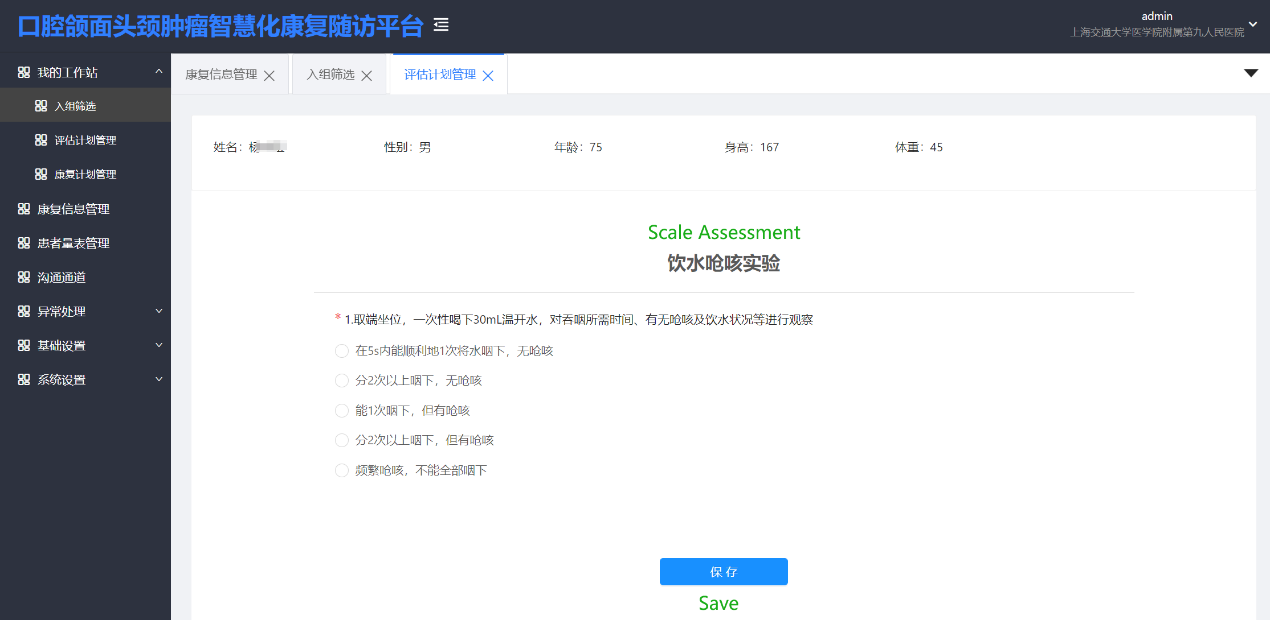
**

- **Programme development**

**
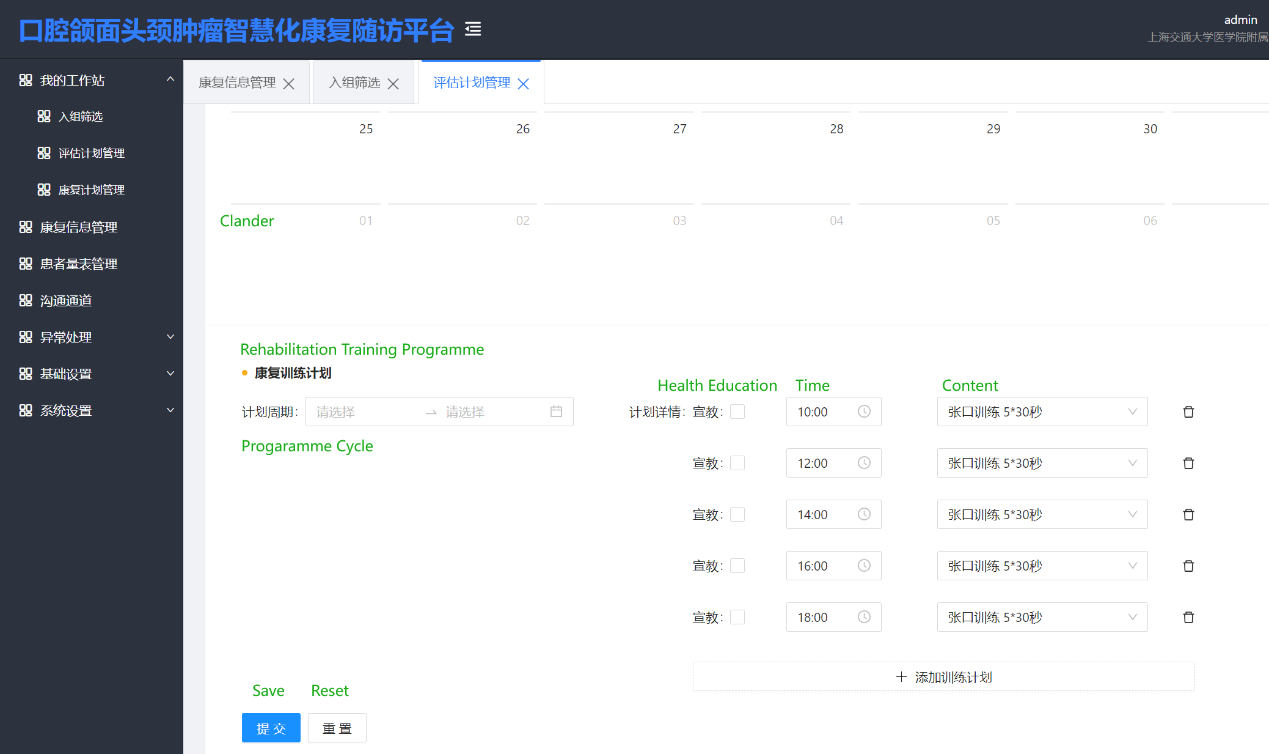
**

- **Flatbed port
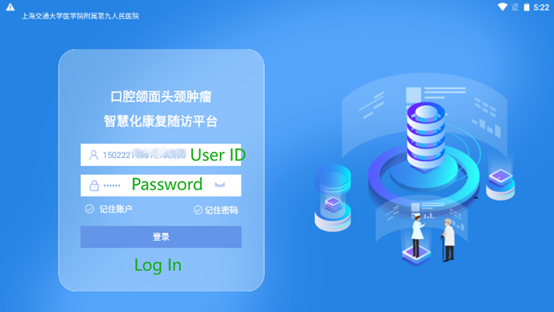
**
- **Main interface**

**
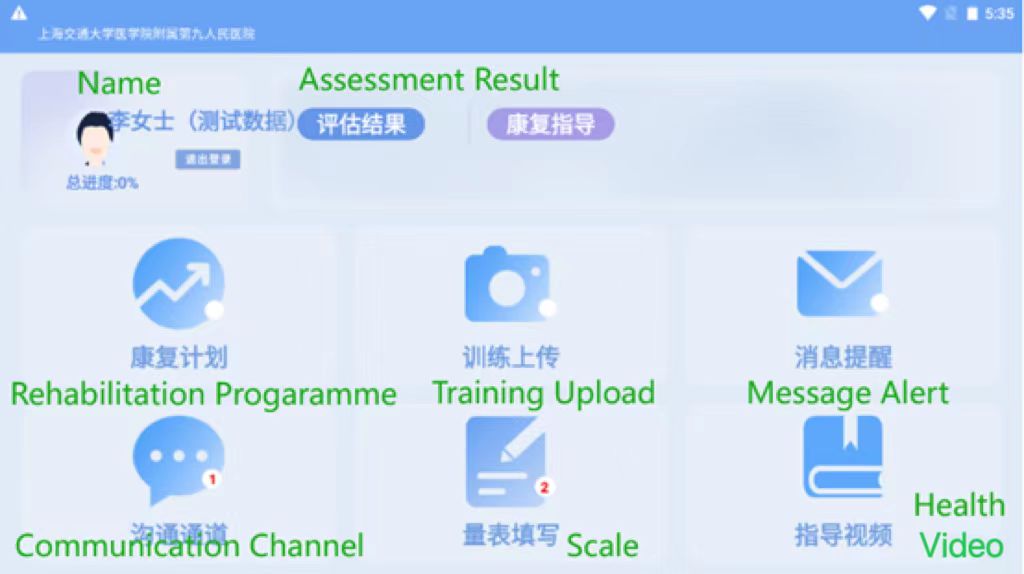
**

- **Rehabilitation Programme**


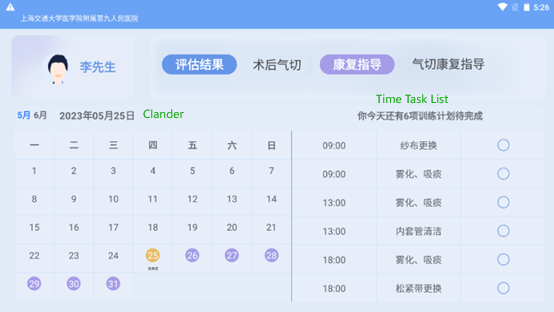


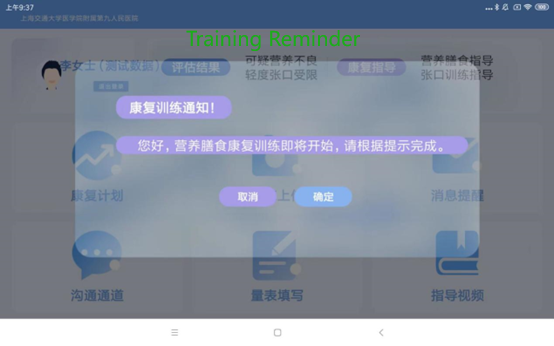


- **Training Upload**


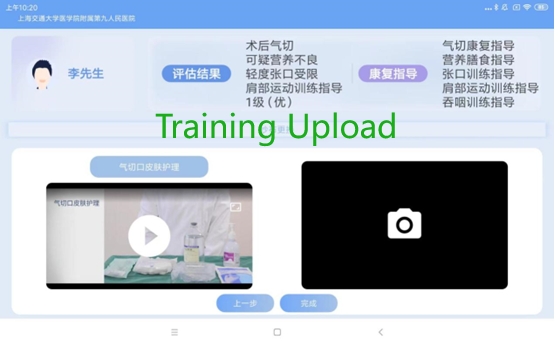


- **Message Alert**


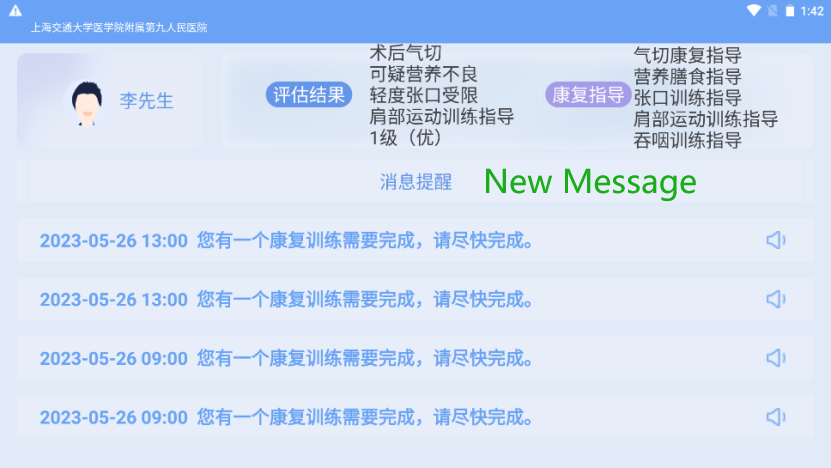


- **Communication Channel**

**
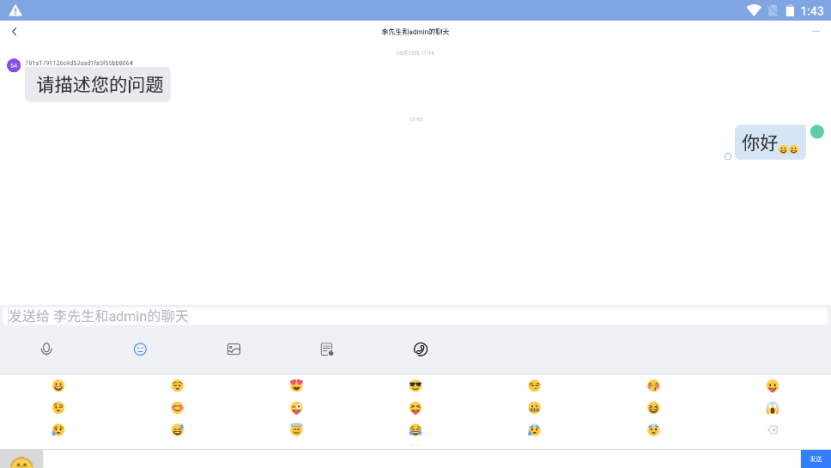
**

- **Scale Management**

**
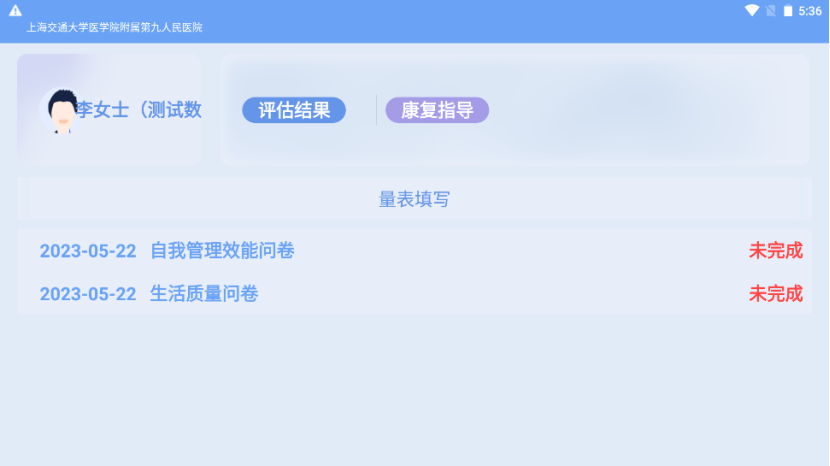
**


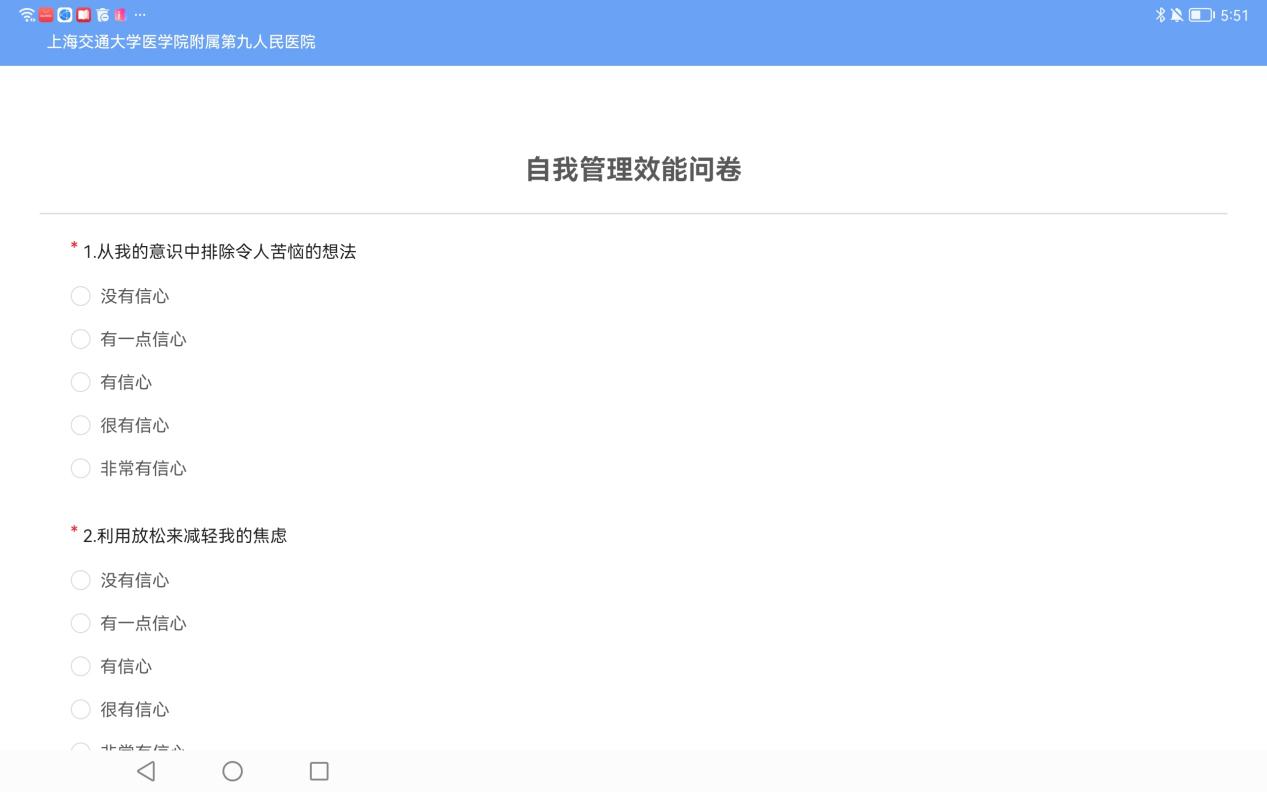


- **Health Education Video**


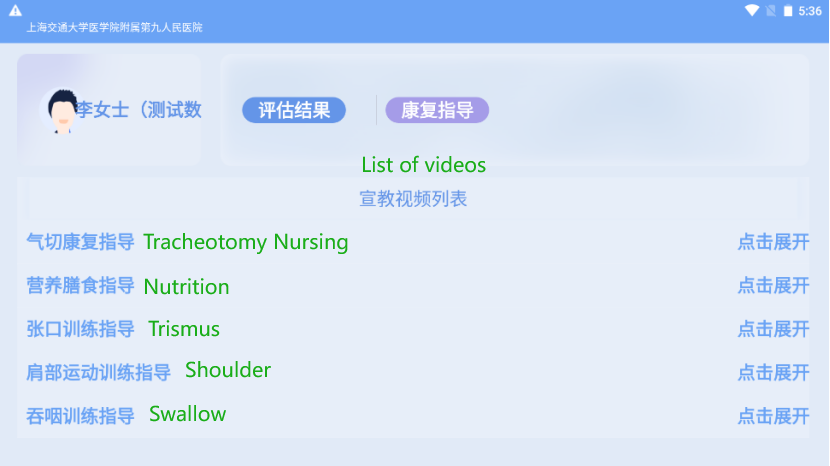


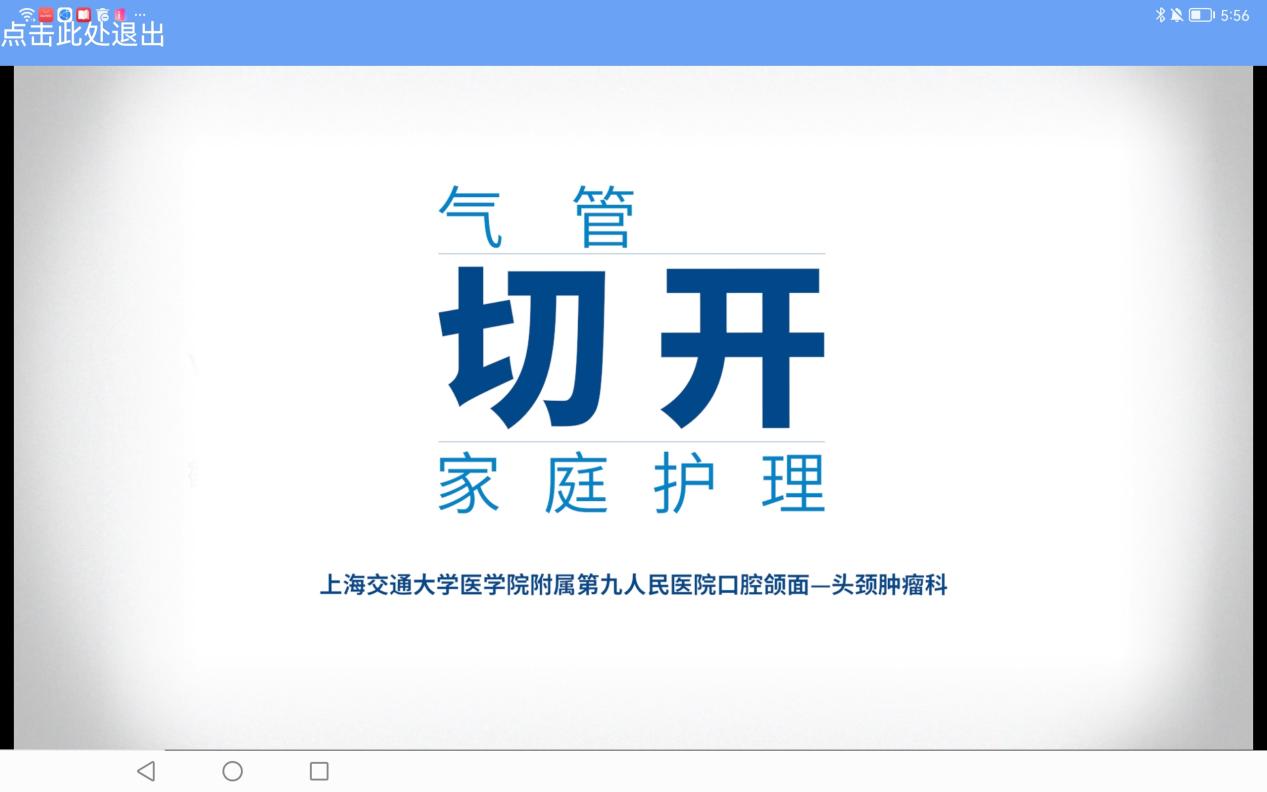

Supplement: Multimedia Appendix 1 [file mhealth-v13-e59926-s001.docx]
