# Supplementary material for: Impact of mHealth on Postoperative Quality of Life, Self-Management, and Dysfunction in Patients With Oral and Maxillofacial Tumors: Nonrandomized Controlled Trial
Source: JMIR Mhealth Uhealth. 2025 Jun 25;13:e59926. doi: 10.2196/59926 (PMC12242703; doi:10.2196/59926)
Supplement: Multimedia Appendix 2 [file mhealth-v13-e59926-s002.docx]

**Table S1.** Participants’ (N=24) Constant-Murley–measured shoulder function scores and repeated-measures ANOVA results before and after the mHealth intervention.

| Variable | T0 | T1 | T2 | Time | Group | Time × Group |
| --- | --- | --- | --- | --- | --- | --- |
|  |  |  |  | F (P value) | | |
| Experimental group, mean (SD) | 60.77 (10.74) | 76.38 (15.20) | 84.46 (8.87) | 112.079 (**<.001**)^a^ | 2.069 (.164) | 0.190 (.667) |
| Control group, mean (SD) | 55.64 (11.15) | 70.09 (13.42) | 77.45 (12.40) |  |  |  |
| t (df) | 1.147 (22) | 1.066 (22) | 1.611 (22) |  |  |  |
| P | .264 | .298 | .121 |  |  |  |

^a^Data in boldface indicate P<.05.

**Table S2.** Participants’ (N=43) water-swallowing test grades before and after the mHealth intervention.

| Time | Group | Grade of water swallow test | | | | | Z value | P value^a^ |
| --- | --- | --- | --- | --- | --- | --- | --- | --- |
|  |  | Ⅰ | Ⅱ | Ⅲ | Ⅳ | Ⅴ |  |  |
| T0 | Control group | 0 | 5 | 3 | 9 | 7 | −0.74 | .46 |
|  | Experimental group | 0 | 5 | 2 | 9 | 3 |  |  |
| T1 | Control group | 3 | 15 | 4 | 0 | 2 | −1.94 | .05 |
|  | Experimental group | 7 | 10 | 1 | 1 | 0 |  |  |
| T2 | Control group | 16 | 6 | 1 | 1 | 0 | −1.00 | .32 |
|  | Experimental group | 15 | 4 | 0 | 0 | 0 |  |  |

^a^Difference between the experimental and control groups.

**Table S3.** Comparison of the improvement effect on dysphagia in the 2 patient groups (N=43).

| Time | Group | Improvement effect on dysphagia | | | | Overall efficiency rate, % | P value^a^ |
| --- | --- | --- | --- | --- | --- | --- | --- |
|  |  | Resolved | Significantly effective | Improved | Ineffective |  |  |
| T1 | Control group | 3 | 9 | 5 | 7 | 70.83 | .12 |
|  | Experimental group | 7 | 6 | 2 | 4 | 78.95 |  |
| T2 | Control group | 16 | 4 | 1 | 3 | 87.50 | .57 |
|  | Experimental group | 14 | 3 | 1 | 1 | 94.74 |  |

^a^Difference between the experimental and control groups.

**Table S4.** Participants’ (N=73) Subjective Objective Management and Analytic grades before and after the mHealth intervention.

| Time | Group | SOMA^a^ grade | | | | | Z value | P value^b^ |
| --- | --- | --- | --- | --- | --- | --- | --- | --- |
|  |  | 0 | Ⅰ | Ⅱ | Ⅲ | Ⅳ |  |  |
| T0 | Control group | 0 | 15 | 15 | 5 | 0 | −1.81 | .86 |
|  | Experimental group | 0 | 17 | 16 | 5 | 0 |  |  |
| T1 | Control group | 7 | 19 | 8 | 1 | 0 | −4.61 | .65 |
|  | Experimental group | 5 | 28 | 5 | 0 | 0 |  |  |
| T2 | Control group | 19 | 13 | 3 | 0 | 0 | −2.01 | .84 |
|  | Experimental group | 20 | 18 | 0 | 0 | 0 |  |  |

^a^SOMA: Subjective Objective Management and Analytic.

^b^Difference between the experimental and control groups.

**Table S5.** Comparison of the improvement effect on trismus in the 2 patient groups (N=73).

| Time | Group | Improvement effect on trismus | | | | Overall efficiency rate, % | P value^a^ |
| --- | --- | --- | --- | --- | --- | --- | --- |
|  |  | Resolved | Significantly effective | Improved | Ineffective |  |  |
| T1 | Control group | 7 | 1 | 14 | 13 | 62.86 | .81 |
|  | Experimental group | 5 | 4 | 14 | 15 | 60.53 |  |
| T2 | Control group | 19 | 2 | 11 | 3 | 91.43 | .53 |
|  | Experimental group | 18 | 4 | 10 | 6 | 84.21 |  |

^a^Difference between the experimental and control groups.
